# Demand spillovers of smash-hit papers: evidence from the ‘Male Organ Incident’

**DOI:** 10.1186/2193-1801-2-168

**Published:** 2013-04-17

**Authors:** Otto Kässi, Tatu Westling

**Affiliations:** University of Helsink and HECER, Helsinki, Finland

**Keywords:** Scholarly spillover, Media, Blogs, Downloads, Natural experiment, Difference in differences, Regression discontinuity design

## Abstract

This study explores the short-run spillover effects of popular research papers. We consider the publicity of ‘Male Organ and Economic Growth: Does Size Matter?’ as an exogenous shock to economics discussion paper demand, a natural experiment of a sort. In particular, we analyze how the very substantial visibility influenced the downloads of Helsinki Center of Economic Research discussion papers. Difference in differences and regression discontinuity analysis are conducted to elicit the spillover patterns. This study finds that the spillover effect to average economics paper demand is positive and statistically significant. It seems that hit papers increase the exposure of previously less downloaded papers. We find that part of the spillover effect could be attributable to Internet search engines’ influence on browsing behavior. Conforming to expected patterns, papers residing on the same web page as the hit paper evidence very significant increases in downloads which also supports the spillover thesis.

**JEL Classification:**

A11, C21

**MSC Classification:**

97K80

**Electronic supplementary material:**

The online version of this article (doi:10.1186/2193-1801-2-168) contains supplementary material, which is available to authorized users.

## Introduction

Economics research papers seldom make headlines. As infrequently do they permeate the online community beyond the academic confines. Something quite unlike was on display in July 2011 following the publication of ‘Male Organ and Economic Growth: Does Size Matter?’ [henceforth MOEG] (Westling 2011) which explored the link between penile length and economic growth^a^. In the weeks that followed it amassed some 175000 downloads and a global coverage in print, television and online media. Tim Harford of *Financial Times* dubbed it the ‘smash-hit economics research paper of the summer’. Arguably the whole incident with all its publicity was completely unanticipated, and came as a surprise to everyone involved. If nothing else, an attractive natural experiment came into being.

Events such as this can be viewed from many angles and disciplines. One intriguing facet is the scholarly visibility that ensued. In particular, it is tempting to speculate whether such ‘hit papers’ generate wider interest on research that emanates from the same institution. At least three motivations are clear. First, academic spillover effects can reveal something about the fabric of scholarly discourse. Second, substantial visibility externalities could alter attractiveness of different publication channels. Third, the incident itself speaks volumes about the impact Internet already has in the academic sphere. On the other hand, the natural experiment character of the event has an obvious appeal from the methodological perspective.

The objective of this study is to explore scholarly spillover effects. Namely, we analyze the download data of Helsinki Center of Economic Research [henceforth HECER] to estimate the impact of the ’male organ incident’ on the short-run demand for the institution’s economics discussion papers – a scholarly spillover effect, if that term is appropriate. The demand shock can be considered exogenous, and indeed the whole incident resembles a natural experiment. The context, therefore, is an attractive venue for causal inference. In a note of caution, however, we remind that the purpose of this study is to only explore the immediate short-run effects. Hence the existence and magnitude of the long-run demand effects remain obscure.

The economic role of blogs in dissemination of research papers is discussed convincingly in McKenzie and Özler (2011). They find very significant peaks in RePEc^b^ visibility [abstracts views and paper downloads] following papers’ coverage in the most influential blogs. Regarding spillover effects, the literature provides supportive findings. In medical research the publicity in the popular media increases citations substantially (Phillips et al. 1991). Somewhat related analyses, concentrating on economics literature, are also provided in Pieters and Baumgartner (2002) and Brown (2003). Ellison (2011) discusses the role of Internet in academic publishing and contains many references of related themes.

Much of the existing literature focuses on the long-term effects, which, of course, might be more important than the immediate impact. Nevertheless, we view the very short-term effects interesting as well. Our view is supported by findings in Edelman and Larkin (2009) who show that researchers seem to manipulate SSRN download statistics to boost their own papers’ visibility.

In this study two datasets and methods are utilized. First, we use a [public] monthly server log that captures itemized download rates for each paper. It contains most research paper series at the University of Helsinki, and hence we are able to form control groups to capture any time fixed effects [FEs]. The data spans a period of 15 months from May 2010 to July 2011. As MOEG went online on the 11th of July and the download activity was at its most intense in the following three weeks, the July data captures the vast majority of the short-term spillover effects. Second, we analyse the raw download log of July 2011. It contains very detailed information of all economics papers’ downloads, and allows us to construct time series of the patterns.

Regression estimations based on difference in differences [DID] methods support the spillover hypothesis. When comparing the downloads in June and July 2011 and allowing for paper FEs, the hit paper effect was positive but not statistically significant – MOEG was found to increase the average downloads of HECER discussion papers by 2 in July. However, when the probability of a paper being downloaded at least once is being looked at, the spillover effect is statistically significant. A hit paper increases this probability by 11%. It thus seems that previously less frequently downloaded papers reap most benefits from the spillover effects. One interpretation is that hit papers broaden institutions’ audience.

Analysis based on regression discontinuity design [RDD] corroborates with previous findings. Depending on specification, MOEG is found to increase the average monthly downloads of economics papers by 0.5 to 1.5. Despite a different estimation method and data, the figure approximates those obtained by DID.

We present evidence that browsing via Internet search engines might capture part of the spillover effect. In fact this study documents a substantial increase in the downloads of papers that appeared on the same web page as MOEG through July. The 4 papers on the same web page experienced an increase of 6 monthly downloads, which is significant at the 5% level. RDD analysis yields similar conclusions: residing on the same web page increase monthly downloads by 6.2 to 7.2.

Quite confidently we conclude that MOEG creates positive spillover effects. The magnitudes might be quantitatively modest but qualitatively interesting nevertheless. However, the 4 papers on the same web page experienced substantial spillover effects.

This paper proceeds as follows. Section ‘Data and estimation’ describes the data and estimation procedures. Section ‘Results’ presents results and section ‘Conclusions’ concludes. The tables and figures are included in the Appendix.

## Data and estimation

The aggregated monthly data is based on library’s public server logs which capture all downloads at a specified time interval^c^ at the University of Helsinki. In total 15 months of data is available. However, due to addition of new papers we mostly use data from June and July 2011. This ensures that the samples of papers in adjacent months are almost equivalent. Concentrating only on two months of data also reduces problems related to autocorrelated error terms which may severely bias our standard error estimates (Bertrand et al. 2004).

A more detailed data would allow to control for papers’ submission dates but is not available. Brief descriptive statistics for June and July 2011 are given in Table 1. It illustrates the skewness of the download patterns, with most papers experiencing only very few downloads during a month.

**Table 1:** **Monthly downloads in June and July 2011, HECER and control groups**

	***June***	***July***
***HECER (n=335)***		
25th percentile	1	3
Median	2	4
75th percentile	4	5.5
Average	3.0	4.7
*Humanities (n=93)*		
25th percentile	0	0
Median	2	4
75th percentile	4	6
Average	8.2	7.3
*Natural sciences (n=870)*		
25th percentile	0	0
Median	0	3
75th percentile	1	5
Average	1.6	3.5

Notes: the figures show the 2011 monthly downloads at different percentiles in the respective series. Downloads of MOEG has been removed from HECER’s July figure.

To explore the spillover effects we employ data of economics discussion papers. As control groups the downloads of natural sciences and humanities papers are used.^d^ If MOEG has a positive demand effect on the control groups, the estimates are biased. In this case the spillover effect on economics papers would be underestimated. However, we find it unlikely that one paper could increase the demand for papers in the fields beyond its own even within the same university. Hence we assume that the spillover is field- but not institution-specific.

The raw download log contains detailed information of all items in the economics discussion paper series for July 2011. Unfortunately for us, the log file does not include HTTP Referer codes which would be needed to study geographical distribution of downloads. Therefore we only have access to date and time of all downloads. To construct time series for each paper, we aggregate the itemized downloads at the day level.

In addition, we attempt to clean downloads by Internet search engines and crawlers. For this, we use the browser field. This is not a complete solution; no amount of clean-up can assure that all crawler related downloads are identified and deleted. Hence to a limited extent they can interfere with our results.

### Between-month estimation

In this section we use data aggregated at the monthly level. The regression models are estimated with OLS using a difference in differences (DID) specification.^e^ In general they have the following functional form

1 $$\begin{array}{l} Q_{i,t}=\beta_{0}+\text{ECO}N_{i}\alpha +\text{MOE}G_{i,t}\delta \\ \;+[\text{ECO}N_{i}*\text{MOE}G_{i,t}]\gamma +\mu_{i,t} \end{array}$$

where *Q*_*i*, *t*_is the number of monthly downloads, *ECON*_*i*_ is a dummy for inclusion in the HECER series, *MOEG*_*i*, *t*_ is the treatment and *i* ∈ {1,…, *N*} denotes individual papers. The parameter of interest is *γ* which identifies the average treatment effect on the paper demand. In order to ensure that paper specific unobservables do not drive our results, we also estimate (1) using paper FEs.^f^

Due to skewed distribution of downloads we also estimate the probability that a paper is downloaded during a month at least *k* times. Hence the specifications alleviate the issue that the average downloads can be distorted by handful of papers that receive very considerable attention. These are estimated by a linear probability model of the form

2 $$\begin{array}{l} \text{Pr}[Q_{i,t}>k]=\beta_{0}+\text{ECO}N_{i}\alpha +\text{MOE}G_{i,t}\delta \\ \;\;+\;[\text{ECO}N_{i}*\text{MOE}G_{i,t}]\gamma +\mu_{i,t}, \end{array}$$

where notation is same as above. In the baseline specification *k* = 0, which is used to estimate the spillover effect’s tendency to change the probability that a given paper is downloaded at least once. Subsequently different values of *k* > 0 are employed to determine the cut-off point at which spillover effects are still observable. The parameter of interest is again *γ*.

### Within-month estimation

In this section we use log data of daily downloads of economics discussion papers. Estimation of the effect of MOEG on daily download patterns is based on RDD.^g^ Three different specification are utilized: baseline, baseline with time trend and baseline with time trend and FEs^h^. The first two are given by

3 $$\;Q_{i,t}=X_{i}^{\prime }\beta +[PG_{i}]휃+\text{MOE}G_{i,t}\gamma +[\text{MOE}G_{i,t}*PG_{i}]\eta +\mu_{i,t}.$$

In our baseline model, *X* includes only a constant and weekend dummies. Our time trend specification supplements the baseline model with a third degree polynomial time trend interacted with the treatment dummy.^i^ Our third alternative specification includes paper fixed effects and weekend dummies.

The parameters of interest are *γ* and *η*, and the former captures the effect of MOEG on the average paper downloads. The latter is the treatment for the 4 papers on the same web page. The treatment MOEG takes place on 15th July, and corresponds to its appearance in Marginal Revolution and Freakonomics. Weekend dummies capture the substantial within-week download volatility.

Due to the rotational behavior of Earth and Helsinki’s location at the GMT+2 time zone, some [local time] Friday [Monday] downloads from Western [Eastern] Hemisphere are recorded at weekends. However, we postulate that these errors largely cancel each other out, and hence that our spillover estimates are insulated by orbital factors.

## Results

We first describe the download profile of MOEG through July 2011. Subsequently OLS regression estimates with DID and RDD specifications are presented. Then the role of Internet search engines is briefly discussed.

**Figure 1:**
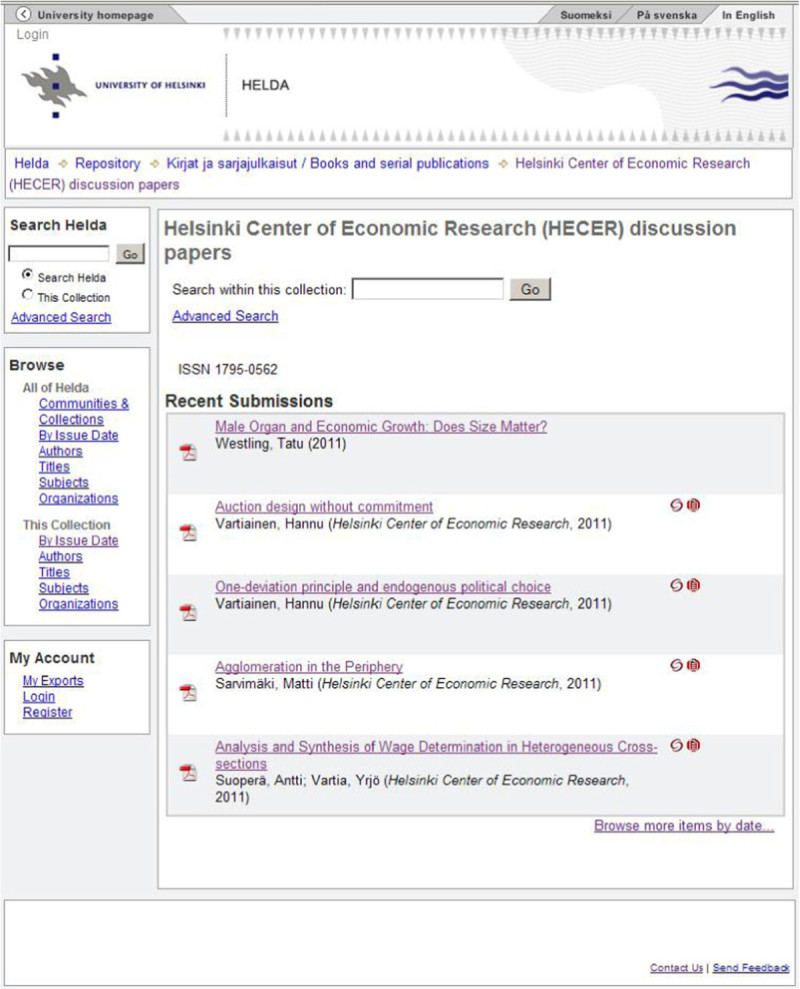
**Screenshot of the discussion paper download web page that appeared throughout July 2011.**

The scale of the exogenous shock can be observed from Figure 1. Moreover, it clarifies the role of Freakonomics and Marginal Revolution in dissemination of papers ^j^. During the 15th July MOEG was mentioned in both blogs, the total downloads ratcheted up from 240 to 5346. One week from there the daily downloads peaked on 22th July at 24410 after which they started to fall. By the end of the July the rate had declined to 750. The total number of MOEG downloads on July was some 175000 which amounts to 60% of all article and paper downloads at the University of Helsinki within the month. Were it not for the Internet, visibility on this scale could not take place. The graph vividly illustrates the effect of blogosphere, Facebook, Twitter and traditional online media combined. Furthermore, the patterns corroborate with the figures presented in McKenzie and Özler (2011). In short, judging from Figure 1 the natural experiment character of the ‘male organ incident’ is evident.

### Spillover effect

The regression estimates with DID specification on monthly downloads in Table 2 suggest that MOEG has a positive if statistically insignificant effect on paper demand. The variable [ECON*MOEG] is found to increase the demand for other discussion papers in HECER series on average by 2. *P*-value for the treatment effect estimate *γ* in pooled OLS DID specification is 0.11. These findings do lend only suggestive support for the spillover thesis.

**Table 2:** **DID estimates on monthly downloads and monthly downloads exceeding zero as dependent variables**

***Dep. variable***	***Monthly dls***		***P***[ ***Monthly dls*** > 0]	
***Model spec.***	***Pooled OLS DID***	***FE DID***	***Pooled OLS DID***	***FE DID***
Constant	6.059***		0.448***	
	(1.062)		(0.016)	
ECON	-3.002.		0.444***	
	(1.082)		(0.024)	
MOEG	-0.334	-0.334	-0.004	-0.004
	(1.263)	(1.087)	(0.017)	(0.023)
ECON*MOEG	1.994	2.018	0.112***	0.113***
	(1.280)	(1.811)	(0.023)	(0.034)
Fixed effects	No	Yes	No	Yes
*R* ^2^	0.0003	0.16	0.20	0.55
N	2525	2525	2525	2525

Notes: Standard errors in parenthesis. Standard errors are clustered on article level. Standard errors of the probability model are corrected for heteroskedasticity. Significance levels in both regressions: *** 0.1%, ** 1%, * 5% and. 10%. Monthly dls refers to monthly downloads.

Controlling for the paper FEs does not materially change the estimate of *γ*. This supports our assumption that the treatment indeed was exogenous and our observations are not driven by paper unobservables.

As can be seen from Table 2, the coefficient of counter-factual [MOEG] at -0.334 is not signicantly different from zero. This suggests that the spillover effect has not contaminated the control groups and verifies our prior that the spillover is field- and not institution-specific.

We are also interested in the broader impact of the hit paper effect. Namely, here the objective is to abstract away the high demand for certain particular papers – which are unlikely to be driven by spillovers – to look whether the majority of papers experience positive demand effects. This is motivated by the fact that idiosyncratic shocks can substantially change demand for very few individual papers. Indeed, the last two columns in Table 2 provide support for the idea that hit papers can increase demand for previously less downloaded papers. MOEG increases the probability that any paper is downloaded during a month by 11%. This coefficient is significant on at least 1% level in both DID specifications. Again paper FEs do not have impact on the qualitative results.

Table 3 provides further support for the idea that hit papers can generate broad interest in institutions’ research. The less downloaded the paper, the higher the relative gain from a spillover. This can be observed from the decreasing *γ* coefficient with respect to cut-off demands *k*. Papers with monthly downloads above 5 evidence on average a 9.2% increase in demand, while more popular papers show very little relative gains. Naturally all values *k* < 5 are highly significant. Consequently it seems that the marginal additions in downloads corresponding to the spillover effect are distributed quite evenly among papers. Indeed there is no *a priori* reason to expect the previously popular papers to receive disproportionate amount of attention. In relative terms, then, the less popular papers gain the most.

**Table 3:** **DID estimates on the probability on monthly downloads exceeding** ***k***

***Cut-off***	Spillover effect
P[Monthly dls > 5]	0.092**
	(0.030)
P[Monthly dls > 10]	0.005
	(0.017)
P[Monthly dls > 15]	0.0002
	(0.014)

Notes: Standard errors are clustered on articles. Standard errors in parenthesis. Significance levels in both regressions: *** 0.1%, ** 1%, * 5% and. 10%. Monthly dls refers to monthly downloads.

Analysis with the RDD specification is aligned with the previous findings. With time trends the estimates in Table 4 imply that the average daily paper demand increases by 0.02 to 0.09 depending on the time bandwidth. These translate to average monthly gains of 0.5 to 1.5, and are roughly in line with the DID estimates^k^. Varying the time bandwidth has only a minor effect on the treatment effect estimates, as do different specifications. Moreover, the coefficient of [MOEG*PG] is largely invariant to changes in specification.

**Table 4:** **RDD estimates on daily downloads in July 2011**

***Dep. variable***		***Daily downloads***	
***Bandwidth***	±15	± 10	±5
*Baseline*			
MOEG	0.021*	0.012	0.043***
	(0.009)	(0.010)	(0.014)
MOEG*PG	0.510***	0.427***	0.366***
	(0.098)	(0.067)	(0.141)
*Polynomial time trend*			
MOEG	0.090***	0.073***	0.090*
	(0.017)	(0.019)	(0.037)
MOEG*PG	0.510***	0.427***	0.366***
	(0.012)	(0.068)	(0.058)
*Article FE*			
MOEG	0.021*	0.012	0.043**
	(0.018)	(0.021)	(0.013)
MOEG*PG	0.511***	0.427***	0.366**
	(0.012)	(0.069)	(0.060)
Robustness check			
*Quasi treatment*	*5th July*	*25th July*	
MOEG	-0.047.	0.062***	
	(0.031)	(0.016)	
MOEG*PG	0.062	0.520***	
	(0.174)	(0.168)	

Notes: All specifications include weekend dummies. The order of the time trend polynomial *f*(*t*) is chosen by AIC. Bandwidth is measured in days around the 15th July. MOEG refers to the 15th July when the paper first appeared on blogs. PG is the same web page dummy. Robustness check with baseline specification and 5 days’ bandwidth. Standard errors in parenthesis. Significance levels: *** 0.1%, ** 1%, * 5% and. 10%.

Quasi treatments are provided as robustness checks. The robustness checks are roughly in line with our main findings: a quasi treatment prior to publication of MOEG is negative and statistically significantly different from zero, but only at 10% level. We suspect that this negative value might be related to a holiday effect: 5th of July in Finland overlaps with July 4th in the United States, which is a national holiday. The later quasi treatment coefficients are largely aligned with our main estimates. We find this reassuring since the spillover effect is likely to exhibit persistence.

### Search engines

**Figure 2:**
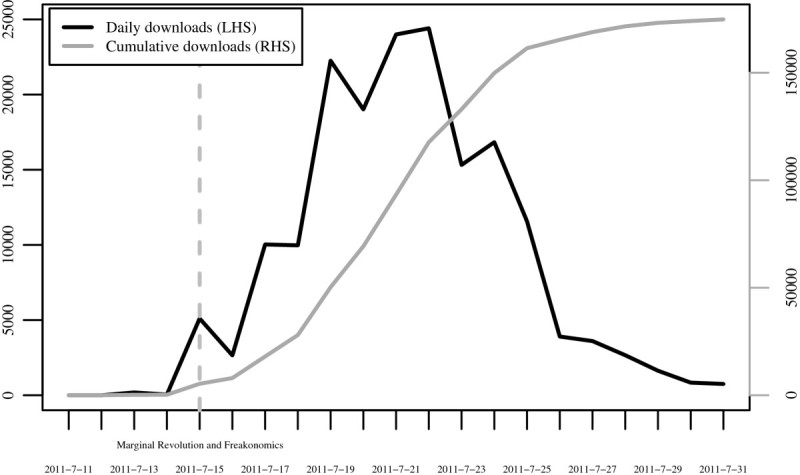
**Daily and cumulative downloads of ‘Male Organ and Economic Growth’.**

It is intriguing to speculate the channels through which the spillover effects operate. Does it rise from ‘genuine interest’ emanating from the sudden institutional visibility? Or does it merely reflect the way Internet search engines drive browsing behavior? To get a clearer picture, Figure 2 presents the window [and the papers] as they appeared to users who browsed to the web page via search engines throughout July 2011. Hence the discussion papers that resided on the same web page as MOEG are known. Since the download statistics of the 4 papers are available, we can compare their rates against typical first and second month figures in the HECER series^l^.

Regression estimates with the difference between the first and second month downloads Table 5. As can be seen [SECOND MONTH*PG], a paper’s appearance on the same page with MOEG through July 2011 induced a higher download activity. The magnitude of this increase is on average 6 downloads, and the coefficient is significant at the 5% level. A note of caution is in order, since seasonal variation could distort the estimation. In particular, if downloads are due to seasonality higher in July than June, then the coefficient [SECOND MONTH*PG] could capture some summer effects. Since substantial part of the download activity most likely stemmed from abroad, it is unlikely that vacations or related factors could seriously compromise the results.

**Table 5:** **DID estimates on downloads between the first and second month after submission**

textitDep. variable	***Monthly downloads***	
***Model spec.***	***Pooled OLS DID***	***FE DID***
Constant	4.153***	
	(0.316)	
SECOND MONTH	-2.255***	-2.283
	(0.448)	(0.339)
PG	10.596***	
	(2.84)	
SECOND MONTH*PG	6.005	6.033*
	(4.016)	(3.034)
Fixed effects	No	Yes
*R* ^2^	0.097	0.485
N	642	642

Notes: Only HECER paper are included. Variable PG corresponds to papers which appeared on the same web page with MOEG through July. SECOND MONTH is by construction the month following the publication. Standard errors in parenthesis. Standard errors are clustered on article level. Significance levels in both regressions: *** 0.1%, ** 1%, * 5% and. 10%.

Analysis with the RDD specification supports previous findings, and is presented in Table 4. It shows that appearing on the same web page increases the daily downloads by 0.365 to 0.51 on average. Translated to monthly figures these correspond to an increase of 6.2 to 7.2 downloads. In the absence of other major exogenous changes – beyond reasonable doubt, that is – we attribute this level shift to the visibility of MOEG. Hence the RDD results presented here support both theses, namely that hit papers generate spillovers and that part of it is driven by search behavior.

## Conclusions

This paper presents evidence of hit papers’ spillover effects by utilizing the demand shock from ‘Male Organ and Economic Growth: Does Size Matter?’ (Westling 2011) as a natural experiment. The paper garnered some 175000 downloads in just three weeks on July 2011, which is a staggering figure by University of Helsinki standards. We explore how the event changed the download patterns of economics research papers at the institution. For robustness, the estimations are conducted both with monthly and daily data, and by utilizing two different analysis methods, namely DID and RDD.

Reflecting on the findings with both approaches, the spillover thesis seems quite robust. Notwithstanding some caveats, it looks as hit papers could increase the demand for research in the short-run. We stress that only RDD results are invariably statistically significant. Depending on the method, the ‘male organ incident’ seems to increase the average monthly downloads by 1.2 to 2. However, the probability that a paper is downloaded at least once during the month increases convincingly, by 11% – hit papers, therefore, entail demand for the previously less exposed research. We interpret this as evidence of a ‘Matthew Effect in Science’, which simply states that high research visibility tends to cumulate to same people and institutions (Merton 1968).

By far the most credible evidence of the spillover effect comes from the within-month analysis. In particular we refer to the papers residing on the same page as MOEG. In monthly figures the incremental downloads reach 6.2 to 7.2. Considerable amount of scepticism is needed to attribute this to chance. We stress, however, that our measures capture only short-term spillover effects.

It must be admitted, though, that the magnitude of the spillover effect is quite modest. Significant amount of publicity is required to generate even a small amount of demand. The numbers imply that on average 0.4% of the 175 000 visitors download research beyond the hit paper. Furthermore, without more detailed log data there is no way to tell how the views are distributed between visitors. On the other hand, the figure of 0.4% might represent the lower bound since only a minor share of the visitors used search engines to locate the paper. Apparently the vast majority came through the direct links of file appearing on blogs and other web pages. The findings presented here lend anecdotal if quite irrefutable support for the prominence of blogs in dissemination of papers, and hence corroborates with the results in McKenzie and Özler (2011). Blogs do matter.

Most importantly, the external validity of the results is somewhat ambiguous. Almost by definition the emergence of a hit paper is a unique event and driven by peculiar circumstances. Whether prospective events yield similar patterns, remains thus unknown.

## Endnotes

^a^ Full disclosure: one author of this paper is the author of MOEG.

^b^ Research Papers in Economics

^c^ The data is available online at https://helda.helsinki.fi/simplestats/front.

^d^ We have chosen humanities and natural sciences as our control group because 1) they are the two largest groups and 2) similar to the economics working paper series the two working paper series consist of preliminary research papers written in English.

^e^ For a text book discussion of DID, see Cameron and Trivedi (2005) pp. 55–57.

^f^ We have also experimented using humanities and science working papers as controls separately. This does not affect the estimates.

^g^ For a text book discussion of RDD see Cameron and Trivedi (2005), pp. 879–893.

^h^ We also estimated a separate model for the 4 papers on the MOEG web page. However, due to the small sample the parameter estimates were insignificant.

^i^ The order of the polynomial is chosen by the Akaike Information Criterion.

^j^ In McKenzie and Özler (2011) Freakonomics, Marginal Revolution, Greg Mankiw, Paul Krugman, The New York Times Economix blog, Dani Rodrik, Chris Blattman and Aid Watch are considered the most influential blogs.

^k^ The average treatment effect on monthly downloads is obtained by multiplying the daily figures by 17. This is the number of days after the publication of MOEG in July.

^l^ The download statistics are aggregated at calendar months. Due to different submission dates within months, the data can be quite noisy. Hence the first month downloads on average represent only 15 days of downloads. However, DID specification accounts for this. We also dropped observations with submission dates on December 2010 and January 2011 since search engines and/or backup procedures added exactly 20 downloads to all papers on the latter month. This January peak can be observed in all papers irrespective of the field or series.

## Authors’ original submitted files for images

Below are the links to the authors’ original submitted files for images. Authors’ original file for figure 1 Authors’ original file for figure 2
