# Supplementary material for: Demand spillovers of smash-hit papers: evidence from the ‘Male Organ Incident’
Source: Springerplus. 2013 Apr 17;2(1):168. doi: 10.1186/2193-1801-2-168 (PMC3685714; doi:10.1186/2193-1801-2-168)
Supplement: Supplementary file 1 — Authors’ original file for figure 1 [file 40064_2012_323_MOESM1_ESM.pdf]

Search Helda
Go
Search Helda
This Collection
Advanced Search

Browse
All of Helda
Communities & Collections
By Issue Date
Authors
Titles
Subjects
Organizations
This Collection
By Issue Date
Authors
Titles
Subjects
Organizations

My Account
My Exports
Login
Register

Helsinki Center of Economic Research (HECER) discussion papers

Search within this collection: Go

Advanced Search

ISSN 1795-0562

Recent Submissions

- Male Organ and Economic Growth: Does Size Matter?
  Westling, Tatu (2011)
- Auction design without commitment
  Vartiainen, Hannu (*Helsinki Center of Economic Research*, 2011)
- One-deviation principle and endogenous political choice
  Vartiainen, Hannu (*Helsinki Center of Economic Research*, 2011)
- Agglomeration in the Periphery
  Sarvimäki, Matti (*Helsinki Center of Economic Research*, 2011)
- Analysis and Synthesis of Wage Determination in Heterogeneous Cross-sections
  Suoperä, Antti; Vartia, Yrjö (*Helsinki Center of Economic Research*, 2011)

Browse more items by date...
