# Supplementary figures and images for: Demand spillovers of smash-hit papers: evidence from the ‘Male Organ Incident’
Source: Springerplus. 2013 Apr 17;2(1):168. doi: 10.1186/2193-1801-2-168 (PMC3685714; doi:10.1186/2193-1801-2-168)

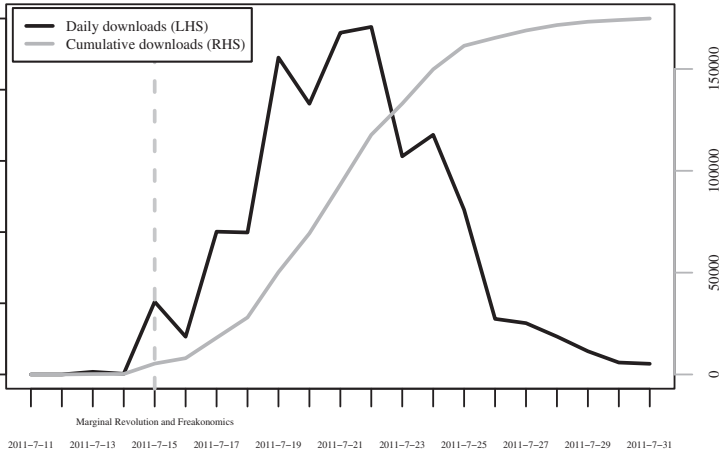

Supplement: Supplementary file 2 — Authors’ original file for figure 2 [file 40064_2012_323_MOESM2_ESM.pdf]
